# Pallidol hexa­acetate ethyl acetate monosolvate

**DOI:** 10.1107/S160053681301708X

**Published:** 2013-06-26

**Authors:** Qinyong Mao, Dennis K. Taylor, Seik Weng Ng, Edward R. T. Tiekink

**Affiliations:** a School of Agriculture, Food and Wine, The University of Adelaide, Waite Campus, PMB 1, Glen Osmond, SA 5064, Australia; b Department of Chemistry, University of Malaya, 50603 Kuala Lumpur, Malaysia; c Chemistry Department, Faculty of Science, King Abdulaziz University, PO Box 80203 Jeddah, Saudi Arabia

## Abstract

The entire mol­ecule of pallidol hexa­acetate {systematic name: (±)-(4b*R*,5*R*,9b*R*,10*R*)-5,10-bis­[4-(acet­yloxy)phen­yl]-4b,5,9b,10-tetra­hydro­indeno­[2,1-*a*]indene-1,3,6,8-tetrayl tetra­acetate} is completed by the application of twofold rotational symmetry in the title ethyl acetate solvate, C_40_H_34_O_12_·C_4_H_8_O_2_. The ethyl acetate mol­ecule was highly disordered and was treated with the *SQUEEZE* routine [Spek (2009 ▶). *Acta Cryst.* D**65**, 148–155]; the crystallographic data take into account the presence of the solvent. In pallidol hexa­acetate, the dihedral angle between the fused five-membered rings (r.m.s. deviation = 0.100 Å) is 54.73 (6)°, indicating a significant fold in the mol­ecule. Significant twists between residues are also evident as seen in the dihedral angle of 80.70 (5)° between the five-membered ring and the pendent benzene ring to which it is attached. Similarly, the acetate residues are twisted with respect to the benzene ring to which they are attached [C—O(carb­oxy)—C—C torsion angles = −70.24 (14), −114.43 (10) and −72.54 (13)°]. In the crystal, a three-dimensional architecture is sustained by C—H⋯O inter­actions which encompass channels in which the disordered ethyl acetate mol­ecules reside.

## Related literature

For synthetic protocols, see: Takaya *et al.* (2005 ▶); Moss *et al.* (2013 ▶). For the spectroscopic characteristics of pallidol hexa­acetate, see: Khan *et al.* (1986 ▶).
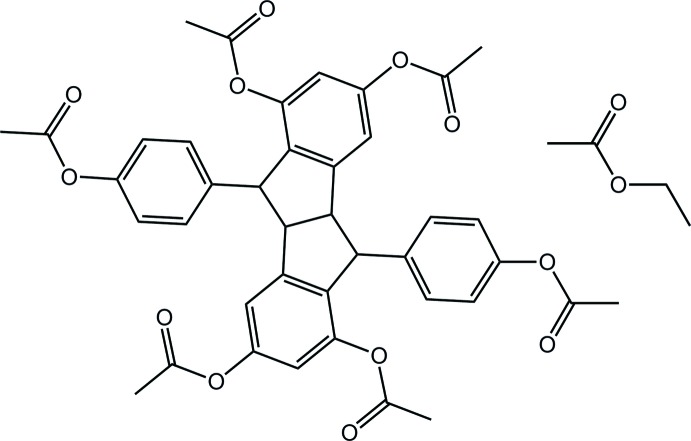


## Experimental

### 

#### Crystal data

C_40_H_34_O_12_·C_4_H_8_O_2_
*M* *_r_* = 794.78
Monoclinic, 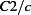

*a* = 13.1495 (1) Å
*b* = 12.7439 (1) Å
*c* = 24.0386 (2) Å
β = 97.186 (1)°
*V* = 3996.65 (5) Å^3^
*Z* = 4
Cu *K*α radiation
μ = 0.83 mm^−1^
*T* = 100 K
0.30 × 0.10 × 0.10 mm

#### Data collection

Agilent SuperNova Dual diffractometer with an Atlas detector
Absorption correction: multi-scan (*CrysAlis PRO*; Agilent, 2013 ▶) *T*_min_ = 0.790, *T*_max_ = 0.922
27173 measured reflections
4029 independent reflections
3714 reflections with *I* > 2σ(*I*)
*R*_int_ = 0.022

#### Refinement

*R*[*F* ^2^ > 2σ(*F* ^2^)] = 0.036
*wR*(*F* ^2^) = 0.095
*S* = 1.02
4029 reflections
238 parameters
H-atom parameters constrained
Δρ_max_ = 0.21 e Å^−3^
Δρ_min_ = −0.22 e Å^−3^

### 

Data collection: *CrysAlis PRO* (Agilent, 2013 ▶); cell refinement: *CrysAlis PRO*; data reduction: *CrysAlis PRO*; program(s) used to solve structure: *SHELXS97* (Sheldrick, 2008 ▶); program(s) used to refine structure: *SHELXL97* (Sheldrick, 2008 ▶); molecular graphics: *ORTEP-3 for Windows* (Farrugia, 2012 ▶) and *DIAMOND* (Brandenburg, 2006 ▶); software used to prepare material for publication: *publCIF* (Westrip, 2010 ▶).

## Supplementary Material

Crystal structure: contains datablock(s) global, I. DOI: 10.1107/S160053681301708X/su2614sup1.cif

Structure factors: contains datablock(s) I. DOI: 10.1107/S160053681301708X/su2614Isup2.hkl

Supplementary material file. DOI: 10.1107/S160053681301708X/su2614Isup3.cml

Additional supplementary materials:  crystallographic information; 3D view; checkCIF report

## Figures and Tables

**Table 1:** Hydrogen-bond geometry (Å, °)

*D*—H⋯*A*	*D*—H	H⋯*A*	*D*⋯*A*	*D*—H⋯*A*
C10—H10⋯O6^i^	1.00	2.51	3.5053 (14)	179
C15—H15⋯O4^ii^	0.95	2.59	3.4834 (12)	158
C20—H20*A*⋯O6^iii^	0.98	2.52	3.2708 (15)	133
C20—H20*B*⋯O2^ii^	0.98	2.28	3.2318 (16)	162

Symmetry codes: (i) 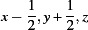; (ii) 
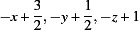
; (iii) 
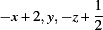
.

**Fig. 1.**
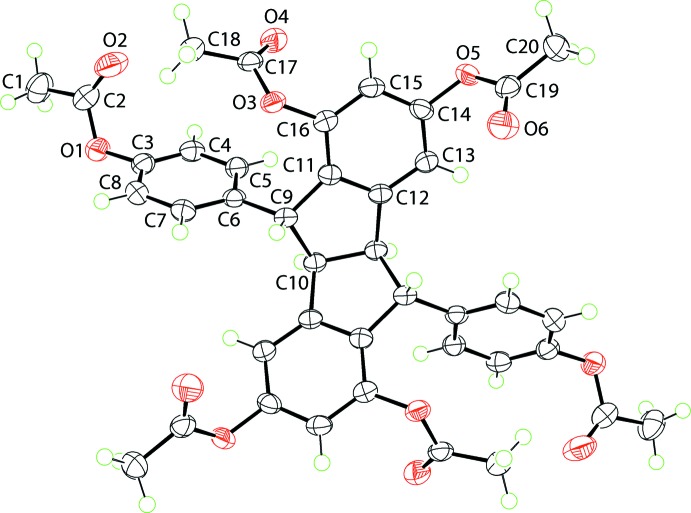
The molecular structure of (I) showing the atom-labelling scheme and displacement ellipsoids at the 50% probability level. Unlabelled atoms are related by the symmetry operation 1 - x, y, 1/2 - z. The disordered ethylacetate molecule is omitted.

**Fig. 2.**
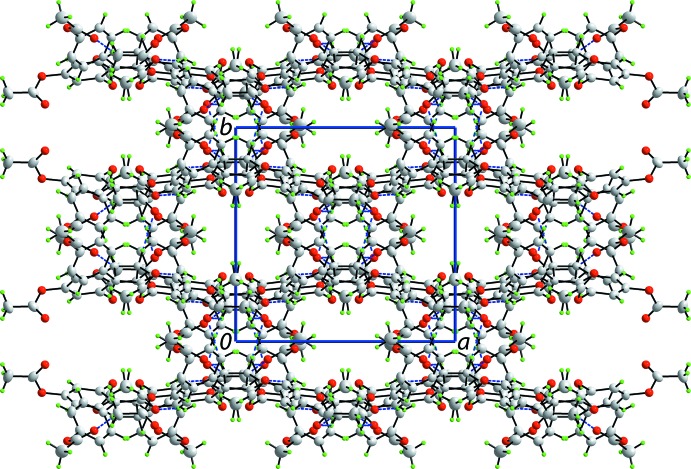
A view in projection down the c axis of the unit-cell contents of (I). The C—H···O interactions are shown as blue dashed lines. The disordered ethylacetate molecules are omitted but, presumably lie in the occupied channels.

## supplementary crystallographic information

### Comment

The synthesis of pallidol hexaacetate was achieved by employing a modified procedure to that reported by Takaya *et al.* (2005) as outlined by Moss *et al.* (2013) which involved the oxidation of *trans*-resveratrol with K_3_Fe(CN)_6_. Herein, the crystal structure determination of pallidol hexaacetate, isolated as its ethylacetate solvate, (I), is described.

The pallidol hexaacetate molecule is disposed about a two-fold rotation axis, Fig. 1. The central five-membered ring is approximately planar with a r.m.s. deviation of 0.100 Å. The maximum deviations = 0.086 (1) Å for the C10 atom and -0.081 (1) Å for the C9 atom, indicating a small twist about the C9—C10 bond. The dihedral angle between the mean planes of the fused five-membered rings is 54.73 (6)°, indicating significant curvature in the molecule. The pendent benzene ring is nearly perpendicular to the mean plane of the five-membered ring to which it is attached, forming a dihedral angle of 80.70 (5)°. None of the acetate residues are co-planar with the benzene ring to which they are attached as seen in the values of the C2—O1—C3—C4 [-70.24 (14)°], C17—O3—C16—C11 [-114.43 (10)°] and C19—O5—C14—C13 [-72.54 (13)°] torsion angles.

In the crystal, molecules assemble into a three-dimensional architecture *via* C—H···O interactions, Fig. 2 and Table 1. In so doing, they define channels in which, presumably, reside the disordered ethylacetate molecules.

### Experimental

To a stirred solution of *trans*-resveratrol (500 mg) and K_2_CO_3_ (245 mg) in MeOH (120 ml) at ambient temperature was slowly added an aqueous solution of K_3_Fe(CN)_6_ (575 mg in 10 ml) over 5 minutes and the mixture stirred for a further 30 minutes. The mixture was then concentrated *in vacuo* and loaded directly onto a flash chromatography column and the organics eluted with EtOAc. The fraction containing the crude dimers was concentrated *in vacuo* and then dissolved in CH_2_Cl_2_ (60 ml) and DMSO (10 ml). Ac_2_O (0.83 ml) and Et_3_N (1.23 ml) were then added and the reaction mixture kept at ambient temperature for 24 h. The reaction was then quenched with NaHCO_3_ (30 ml) and the organics extracted with EtOAc (3 *x* 30 ml). The combined organics were washed with water (20 ml), dried (MgSO_4_) and the volatiles removed *in vacuo*. The acetates were then separated by flash column chromatography (increasing polarity from 20% to 50% ethylacetate in petroleum spirit) to afford pallidol hexaacetate (150 mg) along with several other dimers identified as *trans*-ε-viniferin pentaacetate and *trans*-δ-viniferin pentaacetate (120 mg, 1:2). Recrystallization of the pallidol hexaacetate from neat EtOAc afforded pure material as a colourless crystalline solid. Melting point 486.0–488.4 K; ^1^H NMR (600 MHz, CDCl_3_): δ 7.15 (4*H*, d, J = 8.4 Hz), 7.05 (4*H*, d, J = 8.4 Hz), 6.88 (2*H*, d, J = 1.8 Hz), 6.76 (2*H*, d, J = 1.8 Hz), 4.45 (2*H*, dd, J = 3.0, 3.0 Hz), 4.17 (2*H*, dd, J = 3.0, 3.0 Hz), 2.30 (6*H*, 2 *x* OAc), 2.28 (6*H*, 2 *x* OAc), 1.69 (6*H*, 2 *x* OAc). ^13^C NMR (600 MHz, CDCl_3_): δ 169.46, 169.01 and 167.82 (3 *x* COCH_3_), 150.9, 149.5, 147.9, 147.4, 140.6, 133.6, 128.7, 121.8, 115.4, 115.1, 61.2, 55.7, 21.11, 21.09 and 19.95 (3 *x* COCH_3_). All other spectral data are identical to those previously reported by Khan *et al.* (1986).

### Refinement

C-bound H-atoms were placed in calculated positions and were included in the refinement in the riding model approximation: C—H = 0.95 to 0.98 Å, with *U*_iso_(H) =1.5*U*_eq_(C-methyl) and = 1.2*U*_eq_(C) for other H atoms. The heavily disordered ethyl acetate molecule, lying on a 2-fold rotation axis, was removed by using the SQUEEZE option in *PLATON* (Spek, 2009).

### Crystal data

**Table:**

C_40_H_34_O_12_·C_4_H_8_O_2_	*F*(000) = 1672
*M**_r_* = 794.78	*D*_x_ = 1.321 Mg m^−^^3^
Monoclinic, *C*2/*c*	Cu *K*α radiation, λ = 1.54184 Å
Hall symbol: -C 2yc	Cell parameters from 19786 reflections
*a* = 13.1495 (1) Å	θ = 3.7–74.3°
*b* = 12.7439 (1) Å	µ = 0.83 mm^−^^1^
*c* = 24.0386 (2) Å	*T* = 100 K
β = 97.186 (1)°	Prism, colourless
*V* = 3996.65 (5) Å^3^	0.30 × 0.10 × 0.10 mm
*Z* = 4	

### Data collection

**Table:**

Agilent SuperNova Dual diffractometer with an Atlas detector	4029 independent reflections
Radiation source: SuperNova (Cu) X-ray Source	3714 reflections with *I* > 2σ(*I*)
Mirror monochromator	*R*_int_ = 0.022
Detector resolution: 10.4041 pixels mm^-1^	θ_max_ = 74.4°, θ_min_ = 3.7°
ω scan	*h* = −16→15
Absorption correction: multi-scan (*CrysAlis PRO*; Agilent, 2013)	*k* = 0→15
*T*_min_ = 0.790, *T*_max_ = 0.922	*l* = 0→29
27173 measured reflections	

### Refinement

**Table:**

Refinement on *F*^2^	Primary atom site location: structure-invariant direct methods
Least-squares matrix: full	Secondary atom site location: difference Fourier map
*R*[*F*^2^ > 2σ(*F*^2^)] = 0.036	Hydrogen site location: inferred from neighbouring sites
*wR*(*F*^2^) = 0.095	H-atom parameters constrained
*S* = 1.02	*w* = 1/[σ^2^(*F*_o_^2^) + (0.0505*P*)^2^ + 2.699*P*] where *P* = (*F*_o_^2^ + 2*F*_c_^2^)/3
4029 reflections	(Δ/σ)_max_ = 0.001
238 parameters	Δρ_max_ = 0.21 e Å^−^^3^
0 restraints	Δρ_min_ = −0.22 e Å^−^^3^

### Special details

**Table:**

Geometry. All e.s.d.'s (except the e.s.d. in the dihedral angle between two l.s. planes) are estimated using the full covariance matrix. The cell e.s.d.'s are taken into account individually in the estimation of e.s.d.'s in distances, angles and torsion angles; correlations between e.s.d.'s in cell parameters are only used when they are defined by crystal symmetry. An approximate (isotropic) treatment of cell e.s.d.'s is used for estimating e.s.d.'s involving l.s. planes.

### Fractional atomic coordinates and isotropic or equivalent isotropic displacement parameters (Å^2^)

**Table:**

	*x*	*y*	*z*	*U*_iso_*/*U*_eq_	
O1	0.23221 (6)	0.46511 (7)	0.48061 (3)	0.0295 (2)	
O2	0.35408 (8)	0.41673 (8)	0.54965 (4)	0.0408 (2)	
O3	0.56127 (6)	0.19610 (6)	0.42147 (3)	0.02298 (17)	
O4	0.63350 (6)	0.31813 (7)	0.48240 (3)	0.02886 (19)	
O5	0.88454 (6)	0.23900 (7)	0.35088 (3)	0.02608 (19)	
O6	0.86993 (7)	0.10847 (7)	0.28727 (4)	0.0331 (2)	
C1	0.20807 (11)	0.51331 (11)	0.57260 (5)	0.0374 (3)	
H1A	0.2441	0.5165	0.6108	0.056*	
H1B	0.1442	0.4737	0.5727	0.056*	
H1C	0.1924	0.5846	0.5589	0.056*	
C2	0.27454 (9)	0.46003 (9)	0.53515 (5)	0.0287 (3)	
C3	0.28958 (8)	0.41950 (9)	0.44109 (4)	0.0244 (2)	
C4	0.37948 (8)	0.46632 (9)	0.42947 (4)	0.0238 (2)	
H4	0.4048	0.5278	0.4489	0.029*	
C5	0.43189 (8)	0.42139 (8)	0.38878 (4)	0.0215 (2)	
H5	0.4939	0.4526	0.3805	0.026*	
C6	0.39535 (8)	0.33136 (8)	0.35982 (4)	0.0194 (2)	
C7	0.30498 (8)	0.28622 (9)	0.37288 (4)	0.0238 (2)	
H7	0.2793	0.2248	0.3536	0.029*	
C8	0.25172 (8)	0.32971 (10)	0.41377 (5)	0.0270 (2)	
H8	0.1904	0.2982	0.4227	0.032*	
C9	0.45295 (8)	0.28440 (8)	0.31499 (4)	0.0184 (2)	
H9	0.4240	0.2132	0.3052	0.022*	
C10	0.44581 (7)	0.35182 (8)	0.25998 (4)	0.0185 (2)	
H10	0.4247	0.4252	0.2678	0.022*	
C11	0.56755 (8)	0.27379 (8)	0.33139 (4)	0.0188 (2)	
C12	0.62362 (8)	0.30738 (8)	0.28902 (4)	0.0191 (2)	
C13	0.72954 (8)	0.29618 (8)	0.29446 (4)	0.0218 (2)	
H13	0.7679	0.3192	0.2658	0.026*	
C14	0.77742 (8)	0.25032 (9)	0.34309 (4)	0.0220 (2)	
C15	0.72490 (8)	0.21823 (8)	0.38658 (4)	0.0218 (2)	
H15	0.7599	0.1880	0.4197	0.026*	
C16	0.61939 (8)	0.23177 (8)	0.38003 (4)	0.0207 (2)	
C17	0.57259 (8)	0.24896 (9)	0.47149 (4)	0.0229 (2)	
C18	0.49717 (9)	0.20992 (11)	0.50834 (5)	0.0318 (3)	
H18A	0.5242	0.2217	0.5477	0.048*	
H18B	0.4856	0.1347	0.5019	0.048*	
H18C	0.4322	0.2477	0.4996	0.048*	
C19	0.92348 (9)	0.16363 (9)	0.31875 (4)	0.0255 (2)	
C20	1.03744 (9)	0.16233 (11)	0.32956 (5)	0.0334 (3)	
H20A	1.0641	0.1074	0.3068	0.050*	
H20B	1.0589	0.1480	0.3694	0.050*	
H20C	1.0643	0.2306	0.3197	0.050*	

### Atomic displacement parameters (Å^2^)

**Table:**

	*U*^11^	*U*^22^	*U*^33^	*U*^12^	*U*^13^	*U*^23^
O1	0.0263 (4)	0.0399 (5)	0.0223 (4)	0.0058 (3)	0.0037 (3)	−0.0057 (3)
O2	0.0498 (6)	0.0482 (6)	0.0237 (4)	0.0166 (5)	0.0015 (4)	0.0032 (4)
O3	0.0252 (4)	0.0263 (4)	0.0168 (3)	−0.0011 (3)	0.0004 (3)	0.0028 (3)
O4	0.0302 (4)	0.0337 (4)	0.0223 (4)	−0.0028 (3)	0.0017 (3)	−0.0036 (3)
O5	0.0180 (4)	0.0374 (5)	0.0217 (4)	0.0034 (3)	−0.0021 (3)	−0.0062 (3)
O6	0.0350 (5)	0.0312 (4)	0.0323 (4)	0.0066 (4)	0.0001 (3)	−0.0058 (4)
C1	0.0450 (7)	0.0404 (7)	0.0289 (6)	−0.0006 (6)	0.0127 (5)	−0.0086 (5)
C2	0.0362 (6)	0.0282 (6)	0.0221 (5)	−0.0005 (5)	0.0055 (5)	−0.0006 (4)
C3	0.0235 (5)	0.0311 (6)	0.0185 (5)	0.0061 (4)	0.0020 (4)	−0.0013 (4)
C4	0.0260 (5)	0.0242 (5)	0.0201 (5)	0.0012 (4)	−0.0013 (4)	−0.0026 (4)
C5	0.0219 (5)	0.0220 (5)	0.0202 (5)	−0.0012 (4)	0.0013 (4)	0.0008 (4)
C6	0.0195 (5)	0.0219 (5)	0.0159 (5)	0.0017 (4)	−0.0015 (4)	0.0018 (4)
C7	0.0221 (5)	0.0264 (5)	0.0222 (5)	−0.0031 (4)	−0.0004 (4)	−0.0031 (4)
C8	0.0203 (5)	0.0359 (6)	0.0247 (5)	−0.0022 (4)	0.0025 (4)	−0.0012 (5)
C9	0.0205 (5)	0.0173 (5)	0.0167 (5)	−0.0008 (4)	−0.0009 (4)	0.0004 (4)
C10	0.0205 (5)	0.0172 (5)	0.0172 (5)	0.0009 (4)	0.0003 (4)	0.0005 (4)
C11	0.0206 (5)	0.0165 (5)	0.0186 (5)	0.0005 (4)	0.0002 (4)	−0.0015 (4)
C12	0.0215 (5)	0.0182 (5)	0.0167 (5)	−0.0010 (4)	−0.0011 (4)	−0.0023 (4)
C13	0.0223 (5)	0.0251 (5)	0.0179 (5)	−0.0013 (4)	0.0017 (4)	−0.0034 (4)
C14	0.0179 (5)	0.0260 (5)	0.0209 (5)	0.0014 (4)	−0.0023 (4)	−0.0058 (4)
C15	0.0242 (5)	0.0227 (5)	0.0172 (5)	0.0032 (4)	−0.0025 (4)	−0.0024 (4)
C16	0.0241 (5)	0.0202 (5)	0.0172 (5)	0.0003 (4)	0.0012 (4)	−0.0005 (4)
C17	0.0240 (5)	0.0270 (5)	0.0166 (5)	0.0054 (4)	−0.0014 (4)	0.0028 (4)
C18	0.0305 (6)	0.0432 (7)	0.0220 (5)	0.0002 (5)	0.0043 (4)	0.0066 (5)
C19	0.0282 (6)	0.0289 (6)	0.0193 (5)	0.0082 (4)	0.0026 (4)	0.0045 (4)
C20	0.0254 (6)	0.0429 (7)	0.0321 (6)	0.0093 (5)	0.0044 (5)	0.0073 (5)

### Geometric parameters (Å, º)

**Table:**

O1—C2	1.3603 (14)	C9—C11	1.5152 (14)
O1—C3	1.4103 (13)	C9—C10	1.5697 (13)
O2—C2	1.1953 (15)	C9—H9	1.0000
O3—C17	1.3700 (13)	C10—C12^i^	1.5076 (14)
O3—C16	1.4049 (13)	C10—C10^i^	1.560 (2)
O4—C17	1.1978 (14)	C10—H10	1.0000
O5—C19	1.3712 (13)	C11—C16	1.3854 (14)
O5—C14	1.4050 (13)	C11—C12	1.3972 (14)
O6—C19	1.1955 (14)	C12—C13	1.3900 (15)
C1—C2	1.4941 (16)	C12—C10^i^	1.5076 (13)
C1—H1A	0.9800	C13—C14	1.3857 (15)
C1—H1B	0.9800	C13—H13	0.9500
C1—H1C	0.9800	C14—C15	1.3849 (15)
C3—C8	1.3807 (16)	C15—C16	1.3873 (15)
C3—C4	1.3835 (16)	C15—H15	0.9500
C4—C5	1.3886 (15)	C17—C18	1.4951 (15)
C4—H4	0.9500	C18—H18A	0.9800
C5—C6	1.3957 (15)	C18—H18B	0.9800
C5—H5	0.9500	C18—H18C	0.9800
C6—C7	1.3910 (15)	C19—C20	1.4886 (16)
C6—C9	1.5158 (14)	C20—H20A	0.9800
C7—C8	1.3915 (15)	C20—H20B	0.9800
C7—H7	0.9500	C20—H20C	0.9800
C8—H8	0.9500		
			
C2—O1—C3	116.15 (9)	C12^i^—C10—H10	110.1
C17—O3—C16	117.01 (8)	C10^i^—C10—H10	110.1
C19—O5—C14	115.82 (8)	C9—C10—H10	110.1
C2—C1—H1A	109.5	C16—C11—C12	119.00 (9)
C2—C1—H1B	109.5	C16—C11—C9	128.50 (9)
H1A—C1—H1B	109.5	C12—C11—C9	112.41 (9)
C2—C1—H1C	109.5	C13—C12—C11	120.93 (9)
H1A—C1—H1C	109.5	C13—C12—C10^i^	127.87 (9)
H1B—C1—H1C	109.5	C11—C12—C10^i^	111.20 (9)
O2—C2—O1	122.74 (11)	C14—C13—C12	117.83 (10)
O2—C2—C1	126.19 (11)	C14—C13—H13	121.1
O1—C2—C1	111.06 (10)	C12—C13—H13	121.1
C8—C3—C4	121.86 (10)	C15—C14—C13	122.99 (10)
C8—C3—O1	118.07 (10)	C15—C14—O5	117.09 (9)
C4—C3—O1	120.05 (10)	C13—C14—O5	119.86 (10)
C3—C4—C5	118.44 (10)	C14—C15—C16	117.63 (10)
C3—C4—H4	120.8	C14—C15—H15	121.2
C5—C4—H4	120.8	C16—C15—H15	121.2
C4—C5—C6	121.34 (10)	C11—C16—C15	121.55 (10)
C4—C5—H5	119.3	C11—C16—O3	118.01 (9)
C6—C5—H5	119.3	C15—C16—O3	120.29 (9)
C7—C6—C5	118.55 (10)	O4—C17—O3	123.38 (10)
C7—C6—C9	120.94 (9)	O4—C17—C18	126.17 (10)
C5—C6—C9	120.51 (9)	O3—C17—C18	110.43 (10)
C6—C7—C8	120.95 (10)	C17—C18—H18A	109.5
C6—C7—H7	119.5	C17—C18—H18B	109.5
C8—C7—H7	119.5	H18A—C18—H18B	109.5
C3—C8—C7	118.86 (10)	C17—C18—H18C	109.5
C3—C8—H8	120.6	H18A—C18—H18C	109.5
C7—C8—H8	120.6	H18B—C18—H18C	109.5
C11—C9—C6	114.79 (8)	O6—C19—O5	122.45 (10)
C11—C9—C10	102.76 (8)	O6—C19—C20	127.18 (11)
C6—C9—C10	113.59 (8)	O5—C19—C20	110.37 (10)
C11—C9—H9	108.5	C19—C20—H20A	109.5
C6—C9—H9	108.5	C19—C20—H20B	109.5
C10—C9—H9	108.5	H20A—C20—H20B	109.5
C12^i^—C10—C10^i^	104.29 (9)	C19—C20—H20C	109.5
C12^i^—C10—C9	114.78 (8)	H20A—C20—H20C	109.5
C10^i^—C10—C9	107.37 (8)	H20B—C20—H20C	109.5
			
C3—O1—C2—O2	−2.36 (17)	C10—C9—C11—C12	−11.21 (11)
C3—O1—C2—C1	178.50 (10)	C16—C11—C12—C13	1.96 (15)
C2—O1—C3—C8	111.57 (12)	C9—C11—C12—C13	−174.97 (9)
C2—O1—C3—C4	−70.24 (14)	C16—C11—C12—C10^i^	−179.25 (9)
C8—C3—C4—C5	0.54 (17)	C9—C11—C12—C10^i^	3.83 (12)
O1—C3—C4—C5	−177.59 (9)	C11—C12—C13—C14	0.27 (15)
C3—C4—C5—C6	0.24 (16)	C10^i^—C12—C13—C14	−178.31 (10)
C4—C5—C6—C7	−0.59 (15)	C12—C13—C14—C15	−1.81 (16)
C4—C5—C6—C9	179.16 (9)	C12—C13—C14—O5	−178.90 (9)
C5—C6—C7—C8	0.18 (16)	C19—O5—C14—C15	110.20 (11)
C9—C6—C7—C8	−179.57 (9)	C19—O5—C14—C13	−72.54 (13)
C4—C3—C8—C7	−0.94 (17)	C13—C14—C15—C16	1.04 (16)
O1—C3—C8—C7	177.22 (10)	O5—C14—C15—C16	178.20 (9)
C6—C7—C8—C3	0.57 (17)	C12—C11—C16—C15	−2.79 (15)
C7—C6—C9—C11	−133.77 (10)	C9—C11—C16—C15	173.58 (10)
C5—C6—C9—C11	46.49 (13)	C12—C11—C16—O3	−178.43 (9)
C7—C6—C9—C10	108.39 (11)	C9—C11—C16—O3	−2.06 (16)
C5—C6—C9—C10	−71.36 (12)	C14—C15—C16—C11	1.32 (16)
C11—C9—C10—C12^i^	129.45 (9)	C14—C15—C16—O3	176.87 (9)
C6—C9—C10—C12^i^	−105.94 (10)	C17—O3—C16—C11	−114.43 (10)
C11—C9—C10—C10^i^	14.02 (9)	C17—O3—C16—C15	69.87 (13)
C6—C9—C10—C10^i^	138.64 (8)	C16—O3—C17—O4	−5.04 (15)
C6—C9—C11—C16	48.41 (14)	C16—O3—C17—C18	173.34 (9)
C10—C9—C11—C16	172.23 (10)	C14—O5—C19—O6	−2.25 (15)
C6—C9—C11—C12	−135.02 (9)	C14—O5—C19—C20	178.01 (9)

Symmetry code: (i) −*x*+1, *y*, −*z*+1/2.

### Hydrogen-bond geometry (Å, º)

**Table:**

*D*—H···*A*	*D*—H	H···*A*	*D*···*A*	*D*—H···*A*
C10—H10···O6^ii^	1.00	2.51	3.5053 (14)	179
C15—H15···O4^iii^	0.95	2.59	3.4834 (12)	158
C20—H20*A*···O6^iv^	0.98	2.52	3.2708 (15)	133
C20—H20*B*···O2^iii^	0.98	2.28	3.2318 (16)	162

Symmetry codes: (ii) *x*−1/2, *y*+1/2, *z*; (iii) −*x*+3/2, −*y*+1/2, −*z*+1; (iv) −*x*+2, *y*, −*z*+1/2.
